# Thermography Improves Clinical Assessment in Patients with Systemic Sclerosis Treated with Ozone Therapy

**DOI:** 10.1155/2017/5842723

**Published:** 2017-03-02

**Authors:** Danuta Nowicka

**Affiliations:** Department of Dermatology, Venereology and Allergology, Wrocław Medical University, Wrocław, Poland

## Abstract

*Objective. *Treatment of scleroderma is challenging and limited. The aim of our study was to evaluate the usefulness of thermography in assessment of the clinical condition (joints movability and skin thickness) in clinically advanced patients with systemic sclerosis before and after ozone therapy.* Method. *The study included 42 patients aged 32 to 73 years with advanced systemic sclerosis hospitalized in the university clinic between 2003 and 2006. Thermography and clinical examinations were conducted at baseline and after two series of bath in water with ozone.* Results. *The comparison of results showed significant increase in skin temperature by 2.5°C, significant increase in interphalangeal joints movability by 18 degrees, and significant decrease in skin score by 14.7 points. The skin temperature was correlated with skin score (*r* = −0.59) and joints movability (*r* = +0.8).* Conclusions. *Ozone therapy shows positive effect on clinical parameters and skin temperature as measured with thermography. The study indicated possibility of introducing ozonotherapy as an independent therapy in cases with low level of progression or during remission periods and as additional treatment in patients with advanced disease requiring immunosuppressive treatment. Thermography is useful in assessment of skin condition showing strong correlation between skin temperature and clinical parameters.

## 1. Introduction

Depending on type, dermal changes, and changes in the internal organs constituted the clinical evidence of scleroderma [1, 2]. The dermal changes include indurated edema of the skin and subcutaneous tissue as well as atrophy. The region of distal parts of limbs is initially affected by a doughy edema and erythema, and then the skin becomes numb and fingers become claw-like with limited movability. The sweat and sebaceous glands undergo atrophy. The specific changes affect blood vessels of various sizes; capillary vessels become fibrose with the consequent ischemia, which manifests itself as a decrease in the skin surface temperature. Changes appear in the internal organs as well [3–5].

Treating scleroderma is difficult because its etiology and clinical manifestations are not fully elucidated. The treatment is mainly aimed at limiting the inflammatory processes, slowing down the progression of fibrosis, and reducing the vascular changes. Depending on the phase of illness, the methods most frequently employed are steroid therapy, immunosuppression, penicillamine, UVA I, and vasodilators as well as antioxidants [6–8]. Ozone has a potential to limit the inflammatory processes through the reduction of proliferation of neutrophils and mastocytes, impeding release of acute phase proteins, increasing concentration of prostacyclin 6-keto-PGF1*α*, and decreasing the concentration of prostacyclin PGF2*α*, which results from the effect of oxygen radicals on arachidonic acid. The radicals, which form through the reaction of ozone and short chain fatty acids, are able to displace other oxygen free radicals released during inflammatory, necrotic, and neoplastic processes. Microcirculation in the capillary vessels is improved by making the cell membrane flexible and stable, by limiting the aggregation and adhesion of platelets, owing to the stabilization of disulfide bonds S-S. The main mechanism of activity of the increased concentration of ozone on the organism seems to be associated with its proven activity on stimulating the synthesis of nitric oxide synthase, the enzyme which catalyses the emergence of nitric oxide [9].

The objective of the study was to evaluate the usefulness of thermography in assessment of the clinical condition (joints movability and skin thickness) in clinically advanced patients with systemic sclerosis before and after ozone therapy.

## 2. Materials and Methods

The study was conducted on a group of 42 patients with systemic sclerosis aged 32 to 73 years (mean age: 45 ± 12.7), including 28 women and 14 men hospitalized in the Clinic of Dermatology, Venereology and Allergology, Wrocław Medical University, Poland, between 2003 and 2006. Systemic sclerosis was diagnosed based on clinical presentation, laboratory and radiological examination results, and assessment of the functions of internal organs as well as the examination of capillary vessels using capillaroscopy. In all patients vascular disorders were solely associated with systemic sclerosis. The mean duration of illness was 6.55 ± 5.18 years, ranging from 12 months to 23 years. At the time of the study, patients did not receive any other type of treatment.

The study protocol included the following examinations and procedures:

1. Qualification examination
2. Initial thermography
3. Initial score assessment according to* skin score*
4. Initial assessment of interphalanx joint movability
5. Blood pressure measurement
6. Two series of ten 10-minute comprehensive air baths performed every day separated by 10 days without treatment
7. Control thermography
8. Two series of ten 10-minute comprehensive air baths in water with the mixture of air and oxygen provided in the amount not exceeding 2.42 mg/minute performed every day followed by 10 days without treatment
9. Final thermography
10. Score assessment according to skin score
11. Blood pressure measurement
12. Assessment of interphalanx joint movability

Two series of baths in water without ozone were conducted first to eliminate the influence of physical stimuli on the dilatation of blood vessels during assessment of ozone therapy. A 10-day break between treatments and thermography was used to exclude the influence of confounding factors and determine the stability of the effect of studied treatment.

For baths, the rehabilitation equipment Ozonomatic Jolly Med (Ozonomatic srl, Rome, Italy) was used. While the equipment is in operation, it is possible to adjust the water temperature, the intensity of hydromassage stimulation activity in the range 40–190 mbar, the diameter of bubbles within the range 0.4–1.5 mm, the bath time, and the flow of ozone and air mixture into water. The maximum possible amount of ozone in a stream of air provided to the mat is 2.42 mg/min. In this concentration, ozone dissolves in water and does not reach air above the water level; therefore, there is no toxic activity on the epithelia of the respiratory system of the patient taking the bath and of people operating the equipment.

Thermography was carried out after patient's acclimatization. During thermographic examination with Thermographic Camera V-20 II (VIGO System, Ozarow Mazowiecki, Poland), the analysis of the distribution of temperature of 1/3 distal part of upper limbs (the areas of the wrist, palm back, and fingers) was performed. In order to obtain accurate results of the measurements, the area was marked in the shape of an examined object, temperatures only for this area were analyzed, and thus ambient temperature was eliminated. The examinations were carried out in the room of 24 m^2^ with white walls ensuring constant emissivity, humidity range between 40 and 50%, temperature range between 22 and 24°C, and the presence of only one person who performed the examination. The temperature and humidity were monitored which remained stable. The distance between the examined objects was 80 cm according to producer instructions. The study was conducted during autumn-winter in centrally heating rooms with iron cast radiators emitting constant temperature. During the examinations, the environment conditions were kept unchanged [10].

The preliminary assessment of thermograms was based on reading the color scale where different colors and their hues are assigned different temperature (a lighter hue corresponds to higher temperature while darker hue corresponds to lower temperature). Next, accurate calculations were made using a computer system for temperature value analysis. The results were presented on numerical scale in Celsius degrees.

The progression of systemic sclerosis was assessed with a 0–3 scale (0: normal; 1: mild thickness; 2: moderate thickness; 3: severe thickness) using the Rodman scoring technique adopted by Clement et al. Skin thickness was assessed clinically in terms of the ease of bending skin in a fold in 10 body areas (face, chest, abdomen, back, shoulders, forearms, hands, thighs, shanks, and feet) and the skin score index encompassed the sum of the scores in a total of 17 measurement points [11–13].

The arterial blood pressure was measured using a mercury pressure gauge before beginning the treatment, 30 minutes after each treatment session, and during the control visits after 10 days from the completion of the therapy.

**Figure 1:**
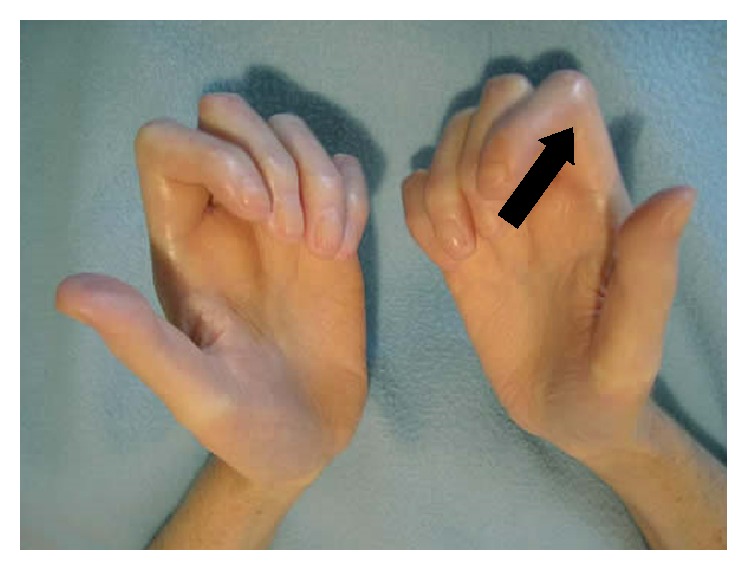
Hands of a female patient suffering from scleroderma with the marked place of the maximum angle of finger opening in proximal interphalanx joint of the dominant hand.

Interphalangeal joint movability examination was carried out with a protractor; the maximum angle of finger opening in proximal interphalanx joints of a dominating hand index finger was assessed [14, 15]. The maximum angle measurement of finger opening is shown in Figure 1.

Data were statistically analyzed with the Statistica software v. 6 (StatSoft, Tulsa, OK, USA). Variables were presented as means, standard deviations, and medians. The dependencies between the results distributed according to the normal distribution were verified using Student's *t*-test and the correlations were analyzed by means of Pearson's correlation coefficient for the dependent groups. The results were considered to be statistically significant at a value of *p* ≤ 0.05.

## 3. Results

**Figure 2:**
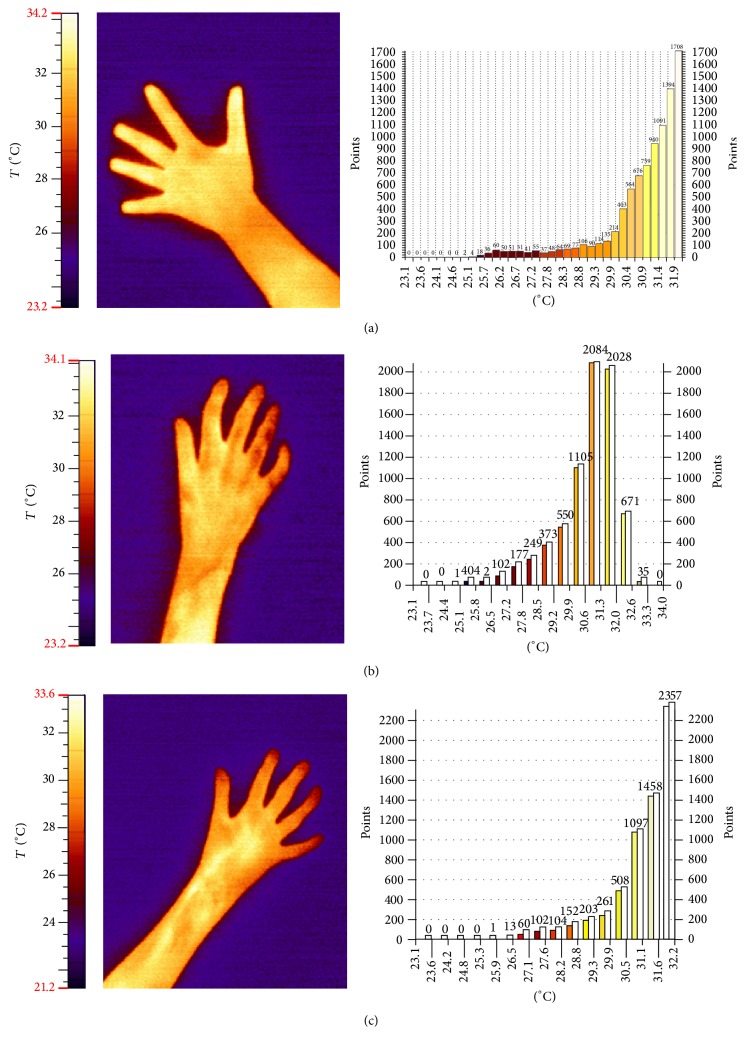
Thermographic pictures with corresponding histogram of color scale. (a) A healthy hand: the noticeably lighter places in the area of distal finger parts and the area of the ball point to higher skin temperature in those places. (b) A hand of a person suffering from systemic sclerosis before ozone therapy: significant lowering of the temperature of phalanx distal parts (especially of the 4th finger) is visible. (c) A hand of a person suffering from scleroderma after ozonotherapy treatments: the increase in the temperature in the entire examined area manifesting itself as lighter colors on the thermogram is visible.

The distribution of temperatures on the entire body of healthy people shows significant deviations, depending on the body part. In healthy skin, blood capillaries are not subjected to the process of fibrosis, so the distal parts of palms are well perfused and warm, which is reflected in the thermographic measurements [16, 17]. The examinations demonstrated that the average temperature of the examined area in healthy patients fluctuates between 29.8 and 33.1°C, depending mostly on the temperature of the environment [18]. The average skin temperature of the palm and distal parts of forearms in the dominant hand of people with systemic sclerosis before the therapy ranged from 23.2 to 27.4°C (mean 25.4°C ± 2.61). In thermograms of those patients, the temperature of distal parts of palm was much lower, as demonstrated by the darker color, while the lighter color correlates with increased perfusion such as the warm middle part of the palm. After two series of ten 10-minute comprehensive baths in water with a mixture of air and ozone, a significant increase in skin surface temperature was achieved (from 25.4 to 29.8°C). Results of thermography examinations in healthy people and patients with systemic sclerosis before and after ozone therapy are shown in Figure 2. In the present study, the results obtained after warm baths without ozone did not change in comparison to baseline values. For this reason only baseline and final results are shown.

The mean skin score index value significantly decreased by 14.7 points; however, no statistical differences between the measurements in particular places in one person were found, which justifies the application of skin score index as a sum of measurement results in 10 areas. The movability of hand joints increased as shown by significant increase by 18 degrees in the angle of the proximal interphalangeal joints of the dominant hand.

Obtaining a measurement of arterial blood pressure in the patients participating in the study was prerequisite for qualifying the patient for each bath. Due to the pathomechanism, the fixed arterial hypertension often coexists with collagenosis, which was confirmed in our study. In the study patients, the mean systolic pressure was 163 ± 15 mmHg and diastolic pressure was 96 ± 10 mmHg. After the treatment, a statistically significant decrease of both systolic and diastolic arterial blood pressure was achieved. The comparison of the results before and after ozone therapy is presented in Table 1.

In order to demonstrate mutual interdependencies between clinical parameters, the correlation coefficient was calculated. It was shown that there exists a positive correlation between the index of scleroderma skin score and the duration of illness as well as the angle of interphalangeal joints and temperature. However, a negative correlation between the index of scleroderma skin score and the temperature of distal part of upper limb and finger movability was found. Analysis of correlations is presented in Table 2.

## 4. Discussion

An important improvement of clinical features of systemic sclerosis such as the increase in the angle of interphalangeal joints, the decrease of skin score index, and the decrease of arterial blood pressure as well as an increase in skin temperature of distal parts of the hand results from the vasodilating effect of ozone, probably through the synthesis of nitric oxide synthase (NOS). The main mechanism of the effect of increased ozone concentration on the organism seems to be associated with its already proven effect on stimulating the synthesis of the enzyme which catalyses the rise of nitric oxide, NOS. Nitric oxide was discovered in 1770 by the chemist Joseph Priestley, but the research on its biochemical importance was initiated much later, in 1977, when it was established that nitric oxide is an active metabolite of compounds dilating blood vessels such as nitroglycerine or sodium nitroprusside. In 1980, it was determined that endothelium lining the blood vessels produces a substance which causes the dilatation of vessel smooth muscles. At that time, this particle was named by the medical, biological, and biochemical experts as endothelium-derived relaxing factor (EDRF). The Nature journal selected it as a particle of 1992 [19, 20].

In 1998, a group of three scientists, Murad, Furchgott, and Ingarro, received the Nobel Prize in medicine for the work (which laid foundations for the discoveries of biological activity of nitric oxide) on the sequence of metabolic changes leading to the generation of this particle from the levorotatory form of the amino acid arginine. L-Arginine by coming into reactions with the reduced form of adenosine diphosphate in oxygen-rich environment generates the levorotatory form of citrulline, water, and nitric oxide. The chemical process catalyst is NOS. Oxygen donor in the reaction taking place during the experiment described in this study is ozone molecule [21].

It has been shown that the chemical structure of nitric oxide synthase (NOS) is not homogenous. In the organism, five isoenzymes of nitric oxide synthase have been distinguished. They are parts of different structure. The brain form (bNOS), also known as cytosol, shows the expression in about 2% of brain neurons as well as in the liver and lungs, the endothelial form (eNOS) appears constitutively in endothelial cells, the hepatocyte form (hepNOS) appears in hepatocytes, and mitochondrial form (mtNOS) is present in all cells. The macrophage form (macNOS)—cytosol, inducible, present in macrophages, Kupffer's cells (liver macrophages), smooth muscles, and chondrocytes—seems to contribute most to the mechanism of ozone baths. It has also been found that macNOS has 50% homology with bNOS, 51% with eNOS, and 82% with hepNOS. It seems that the effect of ozonotherapy on the course of systemic sclerosis depends mostly on the macrophage enzyme type which decides upon the relaxation of the smooth muscular coat of blood vessels. However, the effect through other isoforms of this enzyme cannot be excluded. The sequence which most closely resembles NOS is found in cytochrome reductase P450 (CPR). According to the available research results, the direct activator of NOS is ozone. There are two parallel activated mechanisms: immediate, direct one, which makes the blood vessel muscular coat relax and impedes the adhesion and aggregation of blood platelets, and the long-term one in which phosphorylation mechanisms (most of all S-nitrosylation of cystine proteases) constitute the vasodilating effect of nitric oxide [22–24].

The biological effect of nitric oxide on the blood vessel endothelium is very brief and lasts from a few to a dozen or so seconds; this is due to the deactivation of nitric oxide by hemoglobin immediately after its release from endothelial cells. It is assumed that its effect is of local character and results in the relaxation of the smooth muscular coat of arterial vessels through stimulating activity of guanylyl cyclase and an increase of concentration cyclic GMP. The effect of nitric oxide is synergistic with prostacyclin, especially with reference to impeding the aggregation of blood platelets [20].

The vasodilating effect of nitric oxide has been used in the therapy of sclerosis consisting of an application of pharmacological selective inhibitor of the cyclic form of guanosine monophosphate (cGMP), phosphodiesterase of type 5 (PDE5), that is sildenafil citrate (1-[[3-(6,7-dihydro-1-metylo-7-oxo-3-propyl-1*H*-pyrasolo[4,3-*d*]pyrimidin-5-yl)-4-ethoxyphenyl]sulphonyl]-4-metylopiperasyne) [25, 26]. The effect of the PDE5 activity inhibitor, which produces the disintegration of nitric oxide molecule, is an increase in nitric oxide concentration, causing the blood vessels to become dilated. This mechanism is used in the therapy of hypertension, but the primary indication for using sildenafil is in erectile disorders. In western countries, this compound is used alone or as part of combined therapy for sclerosis and satisfactory results are produced, especially in those patients, in whom pulmonary hypertension develops in the course of the disease. The research confirms the usefulness of the therapy described in this study [26, 27].

The next mechanism of nitric oxide is the reaction of S-nitrosylation of proteins. This reaction takes place with the participation of sulfhydryl groups, one of the most reactive functional groups. The group -SH is most frequently used. It is a component of cysteine side chain and consists of two cystines connected by means of a covalent bond, also known as a disulphide bond. Nitric oxide reacts in the cell with oxygen or metal cations of oxidising characteristics, a nitrosonium ion (NO^+^). This ion is very reactive and bonds with the group -SH of cysteine transforming into the nitrosothiol group -SNO [22, 28].

The following sequence of amino acids predisposing to the nitrosylation reaction (selective nitrosylation) has been observed: XY cysteine (asparagine or glutamine), where X stands for glycine, serine, tryptophan, cysteine, tyrosine, or glutamic acid and Y stands for lysine, arginine, histidine, asparagines, or glutamine. The high affinity of nitric oxide for hemoglobin leads to the production of S-nitrohemoglobin which, after getting into blood vessel endothelium, using similar mechanism to that of potassium channels, creates a reserve of nitric oxide. This amount, with the potential to be gradually released, can account for the prolonged nitric oxide vasodilating effect. This mechanism was observed clinically in the described experiment even 10 days after the direct exposure to the increased concentration of ozone [29, 30].

The objective of this study was to assess the effect of ozonotherapy on the course of systemic sclerosis. It seems that the results obtained in our study prove that applying this type of therapy slows down the progression of the illness by limiting the activation of the immune system which is primarily responsible for the mechanism of fibrosis bringing about clinical changes. The essential property of ozone responsible for its effect on the skin is that it penetrates the epidermis water-fat barrier well and has good solubility in serum. At lower concentrations, this gas does not cause any side-effects connected with the accumulation of strongly reactive oxygen radical O^−^ produced by the disintegration of ozone. In the inhaled air, the concentration limited to below 1 mg/m^3^ does not cause any toxic damage to lung epithelium. In the method under analysis, if total gas solubility in water is assumed, its concentration over the water level would amount to only 0.5 mg/m^3^, which makes it entirely safe both for the patient and for the operating staff.

The procedure model, presented in the study, which consists of two series of ten 10-minute baths daily seems to guarantee positive effect. However, it should be emphasized that working out the therapeutic standard requires follow-up and the analysis of the influence of baths of different duration administered at different frequency. Thus, this calls for further research. Additionally, it should be stressed that this research can be evidence justifying the validity of introducing ozonotherapy as an independent therapy, in cases of conditions of a low level of progression or during remission periods. In more severe conditions with internal organ involvement, the standard immunosuppressive treatment enriched with ozonotherapy should be applied. At present, it is significant that this treatment method is available; the cost of the equipment is low making it an economical choice of therapy.

## 5. Conclusions

Ozone therapy increased the movability of interphalanx joints and decreased the thickness of the skin as well as increasing the superficial skin temperature. Improvement of clinical parameters in patients with advanced systemic sclerosis may be considered an additional treatment modality in this group of patients. Thermography is useful in assessment of skin condition showing strong correlation between skin temperature and clinical parameters.

## Figures and Tables

**Table 1:** The comparison of the results of the analyzed parameters before and after a series of ozonotherapy treatments.

The parameter under analysis	Baseline examination *n* = 42		After ozonotherapy *n* = 42		Statistical significance
	Mean value	Min–max	Mean value	Min–max	*p*
Temperature (°C)	25.4 ± 2.61	23.2–27.4	27.9 ± 3.4	25.4–29.8	0.0042
*Skin score *(points)	42 ± 3.5	33–48	27.3 ± 5.27	19–38	0.0040
The angle of proximal interphalangeal joints (degrees)	142 ± 11	75–170	160 ± 14.5	100–180	0.0002
Systolic pressure (mmHg)	163 ± 15	125–210	130 ± 10	100–155	0.0050
Diastolic pressure (mmHg)	96 ± 10	80–115	88 ± 5	70–110	0.0050

**Table 2:** The comparison of correlations between selected parameters.

	Correlation coefficient	Correlation direction and degree
Skin score, temperature	−0.59	Negative, strong
Skin score, the angle of proximal interphalangeal joints	−0.52	Negative, strong
Skin score before ozonotherapy, the length of illness	+0.18	Positive, weak
The angle of proximal interphalanx joints, temperature	+0.8	Positive, strong

## References

[1] Del Rosso A., Boldrini M., D'Agostino D. (2004). Health-related quality of life in systemic sclerosis as measured by the short form 36: relationship with clinical and biologic markers. *Arthritis Care and Research*.

[2] Hinchcliff M., Varga J. (2008). Systemic sclerosis/scleroderma: a treatable multisystem disease. *American Family Physician*.

[3] Ashida R., Ihn H., Mimura Y. (2009). Clinical and laboratory features of Japanese patients with scleroderma and telangiectasia. *Clinical and Experimental Dermatology*.

[4] Denton C. P., Lapadula G., Mouthon L., Müller-Ladner U. (2009). Renal complications and scleroderma renal crisis. *Rheumatology (Oxford, England)*.

[5] Sulli A., Soldano S., Pizzorni C. (2009). Raynaud's phenomenon and plasma endothelin: correlations with capillaroscopic patterns in systemic sclerosis. *Journal of Rheumatology*.

[6] Comte C., Bessis D., Picot É., Peyron J.-L., Guillot B., Dereure O. (2009). Treatment of connective tissue disorder-related acral syndromes using UVA-1 phototherapy. An open study of 11 cases. *Annales de Dermatologie et de Venereologie*.

[7] Su T.-I. K., Khanna D., Furst D. E. (2009). Rapamycin versus methotrexate in early diffuse systemic sclerosis: results from a randomized, single-blind pilot study. *Arthritis and Rheumatism*.

[8] Denton C. P., Hughes M., Gak N. (2016). BSR and BHPR guideline for the treatment of systemic sclerosis. *Rheumatology*.

[9] Antoszewski L., Moszkowicz T., Kozakiewicz J. (1996). Ozonotherapy in Polish medicine. *Terapia*.

[10] Houdas Y. (1975). Possibilities and limits of infrared thermography. *Lille Medical*.

[11] Black C. M. (1995). Measurement of skin involvement in scleroderma. *Journal of Rheumatology*.

[12] Clements P. J., Hurwitz E. L., Wong W. K. (2000). Skin thickness score as a predictor and correlate of outcome in systemic sclerosis: high-dose versus low-dose penicillamine trial. *Arthritis and Rheumatism*.

[13] Clements P., Lachenbruch P., Siebold J. (1995). Inter and intraobserver variability of total skin thickness score (Modified Rodnan TSS) in systemic sclerosis. *Journal of Rheumatology*.

[14] Brower L. M., Poole J. L. (2004). Reliability and validity of the Duruoz Hand Index in persons with systemic sclerosis (scleroderma). *Arthritis and Rheumatism*.

[15] Clements P. J., Wong W. K., Hurwitz E. L. (1999). Correlates of the disability index of the health assessment questionnaire: a measure of functional impairment in systemic sclerosis. *Arthritis and Rheumatism*.

[16] Howell K. J., Lavorato A., Visentin M. T. (2009). Validation of a protocol for the assessment of skin temperature and blood flow in childhood localised scleroderma. *Skin Research and Technology*.

[17] Riccieri V., Germano V., Alessandri C. (2008). More severe nailfold capillaroscopy findings and anti-endothelial cell antibodies. Are they useful tools for prognostic use in systemic sclerosis?. *Clinical and Experimental Rheumatology*.

[18] Gold J. E., Cherniack M., Hanlon A., Dennerlein J. T., Dropkin J. (2009). Skin temperature in the dorsal hand of office workers and severity of upper extremity musculoskeletal disorders. *International Archives of Occupational and Environmental Health*.

[19] Gryglewski R. J., Botting R. M., Vane J. R. (1988). Mediators produced by the endothelial cell. *Hypertension*.

[20] Palmer R. M. J., Ferrige A. G., Moncada S. (1987). Nitric oxide release accounts for the biological activity of endothelium-derived relaxing factor. *Nature*.

[21] Howlett R. (1998). Nobel award stirs up debate on nitric oxide breakthrough. *Nature*.

[22] Goldstein B. D. (1977). Cellular effects of ozone. *Reviews on Environmental Health*.

[23] Gow A. J., Stamler J. S. (1998). Reactions between nitric oxide and haemoglobin under physiological conditions. *Nature*.

[24] Houpt M. (2009). Using nitrous oxide. *Journal of the American Dental Association*.

[25] Jordan L., Beaver K., Foy S. (2002). Ozone treatment for radiotherapy skin reactions: is there an evidence base for practice?. *European Journal of Oncology Nursing*.

[26] Mathai S. C., Girgis R. E., Fisher M. R. (2007). Addition of sildenafil to bosentan monotherapy in pulmonary arterial hypertension. *European Respiratory Journal*.

[27] Koenig J. Q., Covert D. S., Marshall S. G., van Belle G., Pierson W. E. (1987). The effects of ozone and nitrogen dioxide on pulmonary function in healthy and in asthmatic adolescents. *American Review of Respiratory Disease*.

[28] Laursen B. E., Stankevicius E., Pilegaard H., Mulvany M., Simonsen U. (2006). Potential protective properties of a stable, slow-releasing nitric oxide donor, GEA 3175, in the lung. *Cardiovascular Drug Reviews*.

[29] Chojnowski J., Ponikowska I., Szmurło W. (1998). Clinical studies on the use of ozone baths in the treatment of lower limb ischemia. *Balneologia Poska*.

[30] Ederli L., Morettini R., Borgogni A. (2006). Interaction between nitric oxide and ethylene in the induction of alternative oxidase in ozone-treated tobacco plants. *Plant Physiology*.

